# Orthognathic surgery improves quality of life: a survey clinical study

**DOI:** 10.1186/s12903-024-04638-3

**Published:** 2024-07-25

**Authors:** Esengul Sen, Hatice Duran, Merve Sarı, Nihat Akbulut, Osman Demir

**Affiliations:** 1 Faculty of Dentistry, Department of Oral and Maxillofacial Surgery, Tokat Gaziosmanpasa University, Tokat, Turkey; 2 Faculty of Dentistry, Department of Oral and Maxillofacial Surgery, Kocaeli University, Kocaeli, Turkey; 3 Tokat Oral and Dental Health Center, Tokat, Turkey; 4 Faculty of Dentistry, Department of Oral and Maxillofacial Surgery, Ondokuz Mayis University, Samsun, Turkey; 5 Faculty of Medicine, Department of Biostatistics, Tokat Gaziosmanpasa University, Tokat, Turkey

**Keywords:** Quality of life, Orthognathic surgery, Dentofacial deformities, Questionnaire, Patient satisfaction

## Abstract

**Background:**

The aim of this study was to evaluate the quality of life (QoL) of patients with dentofacial deformity (*n* = 107) compared with that of healthy individuals (*n* = 108) from 2019 to 2020.

**Methods:**

Oral Health Impact Profile 14 (OHIP-14) and the Orthognathic Quality of Life Questionnaire (OQLQ) were administered to the individuals before surgery (T1) and 6 months after surgery (T2).

**Results:**

Preoperative scores (T1) were greater in the surgical group than in the control group in all domains of both surveys (*p* ≤ 0.001). Postoperative scores (T2) in the surgery group decreased significantly after surgery in all domains in both surveys (*p* < 0.001). The OHIP-14 scores in the control group at T2 were significantly greater than those in the other domains except for functional limitation at T1. The type of surgery had no effect on quality of life. Class III patients had higher preoperative scores in certain domains. Postoperative physical disability (*p* = 0.037), physical pain (*p* = 0.047), and preoperative social disability (*p* = 0.030) scores of OHIP-14 awareness of dentofacial aesthetics of OQLQ (*p* = 0.019) were found to be higher in females than in males.

**Conclusions:**

The results showed that orthognathic surgery positively affected quality of life. The control group showed differences in T1 and T2 scores, which can be attributed to their psychological status.

## Background

Dentofacial deformity is an abnormal skeletal malformation that primarily affects the jaw and teeth [1]. Patients with dentofacial deformities have issues with aesthetics, chewing and biting, altered social interactions, and peer pressure to change their appearance. Affected individuals may experience psychological problems and a significant decrease in their quality of life due to these challenges [2–4]. Combining orthodontic and orthognathic treatment procedures can correct dentofacial deformities, which can impact a patient’s quality of life. The World Health Organization (WHO) defines an individual’s quality of life (QoL) as ‘‘perception of their position in life in the context of the culture and value systems in which they live and in relation to their goals, expectations, standards, and concerns’’ [5]. Patients have different motivations for undergoing orthognathic surgery. Some patients expect a better facial appearance, while others expect better oral function, such as biting [6, 7]. Since the primary goal of all treatments is the patient’s well-being, it is essential to understand how treatment affects the patient. Therefore, various scales are used to evaluate the effects of treatment on patients’ quality of life. The Short Form Oral Health Impact Profile Survey (OHIP-14) and Orthognathic Quality of Life Survey (OQLQ) are commonly used tools to assess the quality of life of patients with dentofacial deformities [8–10]. Various studies have evaluated the quality of life of patients who underwent orthognathic surgeries using different surveys and concluded that there were significant improvements in patients’ quality of life after surgery [2, 8, 10–12]. In the literature, studies investigating the effect of traditional orthognathic surgery before and after surgery in control groups using the OHIP-14 and OQLQ are limited [6, 13–16]. Additionally, there are limited studies in the Turkish population [10, 17–19]. Only two of these studies were planned with a healthy control group. Sar et al. investigated the psychosocial and functional outcomes of patients who underwent orthognathic surgery using questionnaires different from those used in our study [19]. Kilinc et al., like our study, investigated the quality of life of patients with only 30 class III deformities treated with orthognathic surgery and 30 healthy controls using the OHIP-14 and OQLQ [10].

This study aimed to investigate the effects of orthognathic surgery on the quality of life of patients with dentofacial deformities compared to those of a healthy control group of dentistry students with standard facial harmony who do not need orthognathic surgery.

We hypothesized that orthognathic surgery would improve patients’ QoL after surgery.

## Methods

### Type of study and study population

A prospective survey study was conducted from 2019 to 2020. The sample size was determined to be 220 participants, which was statistically significant (control group, *n* = 110; surgery group, *n* = 110). Patients who fulfilled the following criteria were included in the surgery group: patients over 18 years of age, those classified as American Society of Anesthesiologists (ASA) classification I, and those who had previously undergone orthodontic treatment for surgery.

The following criteria were excluded: cleft lip and palate, syndromes, facial deformities due to trauma and congenital malformation, pre-existing systemic disease, pregnancy, age < 18 years, previous orthognathic surgery, temporomandibular joint (TMJ) surgery, previous resection due to malignancy in the head-neck region, and previous surgical procedures, such as genioplasty, rhinoplasty, or augmentation during or after orthognathic surgery.

The importance of using healthy people without dentofacial deformities as controls in studies of orthognathic surgery has been highlighted in a systematic review. This is because orthognathic surgery aims to restore facial and dental appearance to that of individuals with normal aesthetic parameters [20]. According to this systematic review, we used healthy individuals in control group. Individuals over 18 years old and classified as American Society of Anesthesiologists (ASA) classification I were included in the control group. Participants in the control group were selected from the younger age group, matching the age of the patients in the surgery group. Because it was reported in studies that orthognathic patients were young [21–23]. Previous orthodontic treatment, complaints about facial appearance, chewing, or symptoms of the temporomandibular joint (TMJ) were considered as exclusion criteria.

Patients in the surgery group underwent orthognathic surgery for jaw deformities. Orthognathic surgeries were performed at two centers, Tokat Gaziosmanpasa University, Department of Oral and Maxillofacial Surgery, and Kocaeli University, Department of Oral and Maxillofacial Surgery, between January 2019 and January 2020.

Initial panoramic films, lateral cephalograms, and computed tomography scans of all patients were obtained after orthodontic treatment before surgery, and patients were divided into groups according to sex, age, type of skeletal anomaly (Classes I, II, III), and surgery type (bimaxillary, single jaw). All patients underwent orthodontic treatment before and after surgery. A Le Fort 1 osteotomy and/or sagittal split osteotomy were performed for each patient according to the case. The patients stayed in the hospital for 3 days, semirigid fixation with plates and screws was performed, and an interocclusal splint was used for two weeks. Subsequently, orthodontic elastics were used, and orthodontic treatment was continued.

### Assessment of health quality

To assess their quality of life, a standard survey was administered to all participants one week before surgery (T1) and six months after surgery (T2). The survey included demographic information and the OHIP-14 and OQLQ instruments. In the surgery group, the surveys were administered after orthodontic treatment before surgery. We conducted surveys for the control group concurrently with the patient group. T1 and T2 refer to the time at which also applied the surveys to the control group at the same time as the patient group.

The OHIP-14 consists of 14 questions containing seven main assessment areas. Questions 1 and 2 represent functional limitations; questions 3 and 4 represent physical pain; questions 5 and 6 represent psychological distress; questions 7 and 8 represent physical disability; questions 9 and 10 represent psychological disability; questions 11 and 12 represent social barriers; and questions 13 and 14 represent handicap areas. The results were evaluated using a five-point Likert scale:0 = never; 1 = rarely; 2 = sometimes; 3 = frequently; and 4 = very often. The results range from 0 to 28 points, with higher scores indicating worse quality of life. This survey was previously translated into Turkish, and its reliability and validity were tested [24].

Cunningham et al. developed a special evaluation survey to measure the QoL of patients with severe skeletal deformities [25]. The original form consists of 22 items. This survey aimed to evaluate the effect of dentofacial deformity on the patients’ quality of life in 4 main areas. These areas are facial aesthetics (items 1, 7, 10, 11, and 14, ranging from 0 to 20), oral function (2 to 6, ranging from 0 to 20), dentofacial aesthetic awareness (8, 9, 12, and 13, ranging from 0 to16), social aspects of dentofacial deformity (items15 to 22, ranging from 0 to 32). These items are also measured on a 5-point Likert scale. 0 points: This sentence does not suit you, or you are not bothered by the dentofacial deformity. 1 point: You are a little uncomfortable, 4 points are very uncomfortable. The results range from 0 to 88 points, and higher scores indicate a worse quality of life. This survey was translated into Turkish by an expert translator. Both forms were prepared in different formats to be answered on the internet.

### Statistical analysis

The data were analyzed with IBM SPSS V23. Kolmogorov-Smirnov test and Shapiro-Wilk test were used to analyze compliance with a normal distribution. Independent samples t-tests were used to compare the scores that conformed to a normal distribution in paired groups, and the Mann-Whitney *U* Test was used to compare the scores that did not conform to a normal distribution. The Wilcoxon test was used to compare the pretest and posttest scores that did not fit the normal distribution in each group. Spearman’s rho correlation coefficient was used to examine the relationship between the ages of the participants and the scale scores that did not fit the normal distribution. The results of the analyses were presented as mean ± standard deviation. The results were considered significant at *p* ≤ 0.05.

## Results

The demographic information of the participants is summarized in Table 1.

A total of 215 participants completed the survey (control group, *n* = 108; surgery group, *n* = 107). Five participants did not complete the surveys. In the surgery group, there was a significant decrease in postoperative OHIP-14 (Table 2) and OQLQ scores (Table 3) (*p* ≤ 0.001). The OHIP-14 scores of the surgery group were greater than those of the control group at T1 in all the surveys (*p* < 0.001). The OQLQs for the oral function domain were greater in the surgery group at T2 than in the control group, and the postoperative scores for the aesthetic awareness of the dentofacial and facial aesthetic domains were significantly lower in the surgery group than in the control group (*p* < 0.001). The OQLQ scores at T2 were greater than the scores at T1 in the domains of dentofacial aesthetic awareness (*p* < 0.001) and facial aesthetics (*p* < 0.001) in the control group (Table 3).

**Table 1:** Demographic characteristics of the study participants

	Control Group *n* (%)	Surgery Group *n* (%)
**Age (Mean)**	24.3	24.1
Female	24.4	26.0
Male	24.1	22.8
**Sex** Female Male Total	63 (57%) 45 (43%) 108	61 (57%) 46 (43%) 107
**Type of deformity** Cl I Cl II Cl III	0 0 0	- 17(16%) 90 (84%)
**Type of Surgery** Single jaw Double jaw	0 0	32 (30%) 75 (70%)

**Table 2:** OHIP-14 scores according to the groups

	Groups		p*
	Control Group (n = 108)	Surgery Group (n = 107)	
	Mean ± Std.Dev	Mean ± Std.Dev	
OHIP-14 (T1)	4.39 ± 5,87	22.46 ± 10.46	**< 0**,**001**
OHIP-14 (T2)	9.19 ± 7.5	10.36 ± 8.52	0.417
p**	**< 0.001**	**< 0.001**	
Functional limitation (T1)	0.48 ± 0.99	2.52 ± 2.08	**< 0.001**
Functional limitation (T2)	0.39 ± 0.97	1.47 ± 1.57	**< 0.001**
p**	0.394	**< 0.001**	
Physical pain (T1)	1.19 ± 1,3	3.51 ± 2.06	**< 0.001**
Physical pain (T2)	1.94 ± 1.91	2.49 ± 1.99	**0.030**
p**	**0.004**	**< 0.001**	
Psychological discomfort (T1)	0.72 ± 1.16	4.51 ± 2.18	**< 0.001**
Psychological discomfort (T2)	2.91 ± 1.62	2.07 ± 2.05	**< 0.001**
p**	**< 0.001**	**< 0.001**	
Physical disability (T1)	0.54 ± 0.9	2.87 ± 2.19	**< 0.001**
Physical disability (T2)	0.94 ± 1.44	1.42 ± 1.65	**0.025**
p**	**0.022**	**< 0.001**	
Psychological disability (T1)	0.6 ± 0.97	3.81 ± 2.07	**< 0.001**
Psychological disability (T2)	1.38 ± 1.78	1.11 ± 1.42	0.524
p**	**< 0.001**	**< 0.001**	
Social disability (T1)	0.47 ± 1.01	2.97 ± 2.04	**< 0.001**
Social disability (T2)	0.99 ± 1.43	0.98 ± 1.58	0.752
p**	**0.001**	**< 0.001**	
Handicap (T1)	0.38 ± 1.08	2.25 ± 1.74	**< 0.001**
Handicap (T2)	0.66 ± 1.04	0.81 ± 1.3	0.566
p**	**0.010**	**< 0.001**	

*p* < 0.05 is significant p*, Differences between the groups; p**, Differences between T1 and T2

Variables are presented as the mean ± standard deviation

*Mann Whitney U Test; **Wilcoxon Test

**Table 3:** OQLQ scores according to the groups

	Groups		p*
	Control (*n* = 108)	Surgery (*n* = 107)	
	Mean ± Std.Dev	Mean ± Std.Dev	
OQLQ scores (T1)	11.87 ± 12.38	44.14 ± 18.71	**< 0.001**
OQLQ scores (T2)	25.73 ± 10.91	18.1 ± 16.37	**< 0.001**
p**	**< 0.001**	**< 0.001**	
Facial Aesthetics (T1)	3.25 ± 3.5	11.02 ± 5.19	**< 0.001**
Facial Aesthetics (T2)	9.74 ± 3.27	4.06 ± 4.28	**< 0.001**
p**	**< 0.001**	**< 0.001**	
Oral Function (T1)	2.08 ± 3.17	9.11 ± 5.01	**< 0.001**
Oral Function (T2)	3 ± 3.21	5 ± 4.85	**0.004**
p**	**0.012**	**< 0.001**	
Dentofacial Aesthetic Awareness (T1)	2.92 ± 3.15	8.2 ± 4.17	**< 0.001**
Dentofacial Aesthetic Awareness (T2)	8.41 ± 3.9	4.49 ± 3.93	**< 0.001**
p**	**< 0.001**	**< 0.001**	
Social Aspects of Dentofacial Deformity (T1)	2.79 ± 4.32	12.14 ± 6.2	**< 0.001**
Social Aspects of Dentofacial Deformity (T2)	3.58 ± 3.76	3.54 ± 4.88	0.175
p**	0.050	**< 0.001**	

*p* < 0.05 is significant p*, Differences between the groups; p**, Differences between T1 and T2

Variables are presented as the mean ± standard deviation

*Mann Whitney U test; **Wilcoxon test

When the data from the surgery group were evaluated in terms of sex, the postoperative physical disability (*p* = 0.037), physical pain (*p* = 0.047), and preoperative social disability (*p* = 0.030) scores of the OHIP-14 awareness of dentofacial aesthetics on the OQLQ (*p* = 0.019) were found to be greater in females than in males (Table 4). During the postoperative period, males and females had similar scores. The scores for the facial aesthetics (*p* = 0.008) and dentofacial aesthetic awareness (*p* = 0.002) domains at T1 were significantly greater in females than in males in the control group (Table 4). At T2, there were no differences in the scores.

**Table 4:** OHIP-14 and OQLQ scores according to the groups in the terms of sex

OHIP-14	Sex				p1	p2
	Female		Male			
	Surgery Group (*n* = 61) Mean ± Std.Dev	Control Group (*n* = 63) Mean ± Std.Dev	Surgery Group (*n* = 46) Mean ± Std.Dev	Control group (*n* = 45) Mean ± Std.Dev		
Functional limitation (T1)	2.38 ± 2.02	0.43 ± 1.01	2.73 ± 2.17	0.54 ± 0.96	0.443**	0.551**
Functional limitation (T2)	1.48 ± 1.52	0.46 ± 1.13	1.45 ± 1.66	0.28 ± 0.69	0.756**	0.348**
Physical pain (T1)	3.68 ± 1.91	1.25 ± 1.38	3.27 ± 2.26	1.09 ± 1.19	0.175**	0.51**
Physical pain (T2)	2.79 ± 1.99	2.11 ± 2.17	2.05 ± 1.92	1.65 ± 1.45	0.047**	0.216**
Psychological discomfort (T1)	4.78 ± 2.24	0.43 ± 0.82	4.14 ± 2.05	0.67 ± 0.99	0.093**	0.173**
Psychological discomfort (T2)	2.3 ± 2.13	2.79 ± 1.75	1.75 ± 1.92	3 ± 1.48	0.183**	0.518**
Physical disability (T1)	3.19 ± 2.24	0.43 ± 0.82	2.41 ± 2.04	0.67 ± 0.99	0.080**	0.173**
Physical disability (T2)	1.7 ± 1.74	1 ± 1.56	1.02 ± 1.45	0.83 ± 1.25	0.030**	0.534**
Psychological disability (T1)	4.03 ± 1.96	0.57 ± 1.01	3.5 ± 2.2	0.63 ± 0.9	0.139**	0.754**
Psychological disability (T2)	1.21 ± 1.54	1.38 ± 1.9	0.98 ± 1.23	1.35 ± 1.62	0.552**	0.924**
Social disability (T1)	3.3 ± 2.08	0.48 ± 1.12	2.5 ± 1.92	0.46 ± 0.84	0.043**	0.92**
Social disability (T2)	1.11 ± 1.58	0.97 ± 1.48	0.8 ± 1.58	1 ± 1.37	0.238**	0.909**
Handicap (T1)	2.24 ± 1.52	0.3 ± 1.13	2.27 ± 2.04	0.48 ± 1.01	0.630**	0.401**
Handicap (T2)	0.89 ± 1.43	0.73 ± 1.19	0.7 ± 1.09	0.54 ± 0.78	0.607**	0.327**
**OQLQ**						
Oral Function (T1)	9.83 ± 4.82	2.11 ± 2.62	8.09 ± 5.15	2 ± 3.81	0.051**	0.857**
Oral Function (T2)	5.51 ± 4.93	3.27 ± 3.67	4.27 ± 4.68	2.62 ± 2.41	0.161**	0.304**
Facial Aesthetics (T1)	11.75 ± 4.75	4.37 ± 4.6	9.98 ± 5.66	2.2 ± 2.44	0.080**	0.002**
Facial Aesthetics (T2)	4.32 ± 4.02	2.11 ± 2.62	3.68 ± 4.65	2 ± 3.81	0.149**	0.857**
Dentofacial Aesthetic Awareness (T1)	8.98 ± 4.09	3.7 ± 3.47	7.07 ± 4.06	2.17 ± 2.44	0.019*	0.008*
Dentofacial Aesthetic Awareness (T2)	4.63 ± 3.97	8.73 ± 4.06	4.27 ± 3.9	7.96 ± 3.66	0.639**	0.311**
Social Aspects of Dentofacial Deformity (T1)	13.13 ± 5.96	3.6 ± 5.34	10.73 ± 6.32	2.65 ± 5.18	0.087**	0.355**
Social Aspects of Dentofacial Deformity (T2)	3.83 ± 5.01	3.27 ± 3.26	3.14 ± 4.72	4.02 ± 4.37	0.347**	0.307**

p1, Differences between females and males in the surgery group

p2, Differences between females and males in the control group

Variables are presented as the mean ± standard deviation

*Mann Whitney U test; **Wilcoxon test

The type of surgery (single or double jaw) had no significant effect on the QoL of the patients, according to either the preoperative or postoperative scores (Table 5).

Class III patients had significantly greater scores for OHIP-14 physical pain (*p* = 0.035), social disability (*p* = 0.044), oral function (*p* = 0.026), and dentofacial aesthetic awareness (*p* = 0.034 ) for the OQLQ before surgery. Patients with Cl II and Cl III had similar postoperative OHIP-14 and OQLQ scores in all the domains (Table 5).

**Table 5:** OHIP-14 and OQLQ scores according to surgery type and skeletal anomaly

	Type of Surgery		p1	Skeletal Anomaly		p2
	Single- Jaw Surgery (*n* = 32)	Double-Jaw Surgery (*n* = 75)		Class 2 (*n* = 17)	Class 3 (*n* = 90)	
	Median ± Std.Dev	Median ± Std.Dev		Median ± Std.Dev	Median ± Std.Dev	
OHIP-14 (T1)	20.79 ± 9.99	23.2 ± 10.65	0.272*	17.35 ± 11.25	23.42 ± 10.09	0.028*
OHIP-14 (T2)	10.15 ± 8.27	10.45 ± 8.68	0.906**	11.59 ± 11.9	10.12 ± 7.79	0.983**
Functional limitation (T1)	2.15 ± 1.91	2.69 ± 2.14	0.251**	1.71 ± 1.76	2.68 ± 2.11	0.080**
Functional limitation (T2)	1.42 ± 1.73	1.49 ± 1.51	0.591**	1.59 ± 1.5	1.44 ± 1.59	0.566**
Physical pain (T1)	3.27 ± 1.74	3.62 ± 2.19	0.432**	2.53 ± 1.59	3.7 ± 2.09	0.035**
Physical pain (T2)	2.42 ± 2.29	2.51 ± 1.85	0.582**	2.35 ± 2.15	2.51 ± 1.97	0.704**
Psychological discomfort (T1)	4.24 ± 2.31	4.64 ± 2.12	0.412**	3.82 ± 2.46	4.64 ± 2.11	0.214**
Psychological discomfort (T2)	1.91 ± 2.04	2.15 ± 2.07	0.592**	2.88 ± 2.45	1.92 ± 1.95	0.115**
Physical disability (T1)	2.64 ± 2.21	2.97 ± 2.18	0.485**	2.18 ± 2.38	3 ± 2.14	0.120**
Physical disability (T2)	1.52 ± 1.66	1.38 ± 1.66	0.646**	1.41 ± 1.97	1.42 ± 1.6	0.683**
Psychological disability (T1)	3.61 ± 2.08	3.91 ± 2.08	0.482**	3.06 ± 2.25	3.96 ± 2.02	0.147**
Psychological disability (T2)	1.12 ± 1.29	1.11 ± 1.48	0.742**	1.41 ± 2	1.06 ± 1.28	0.584**
Social disability (T1)	2.88 ± 1.95	3.01 ± 2.1	0.748**	2.06 ± 2.3	3.14 ± 1.96	0.044**
Social disability (T2)	1 ± 1.48	0.97 ± 1.63	0.770**	1 ± 1.8	0.98 ± 1.54	0.901**
Handicap (T1)	2 ± 1.58	2.36 ± 1.81	0.319**	2 ± 1.7	2.3 ± 1.76	0.418**
Handicap (T2)	0.76 ± 1.17	0.84 ± 1.36	0.820**	0.94 ± 2.01	0.79 ± 1.13	0.645**
OQLQ (T1)	43.7 ± 18.36	44.34 ± 18.98	0.871*	34.41 ± 20.18	45.98 ± 17.94	0.019*
OQLQ (T2)	18.85 ± 15.99	17.77 ± 16.63	0.539**	20.29 ± 21.63	17.69 ± 15.3	0.939**
Facial Aesthetics (T1)	10.97 ± 5.12	11.04 ± 5.26	0.987**	8.94 ± 5.21	11.41 ± 5.12	0.083**
Facial Aesthetics (T2)	4.52 ± 4.72	3.85 ± 4.09	0.583**	4.71 ± 4.98	3.93 ± 4.16	0.502**
Oral Function (T1)	8.97 ± 5.2	9.18 ± 4.96	0.845*	6.47 ± 4.96	9.61 ± 4.89	0.026**
Oral Function (T2)	4.64 ± 5.21	5.16 ± 4.7	0.374**	4.94 ± 5.2	5.01 ± 4.81	0.864**
Dentofacial Aesthetic Awareness (T1)	8.52 ± 3.99	8.05 ± 4.27	0.561**	7.29 ± 4.3	8.37 ± 4.15	0.333*
Dentofacial Aesthetic Awareness (T2)	4.45 ± 3.5	4.5 ± 4.13	0.794**	5 ± 4.56	4.39 ± 3.82	0.754**
Social Aspects of Dentofacial Deformity (T1)	11.91 ± 5.31	12.24 ± 6.59	0.800**	9.24 ± 6.23	12.69 ± 6.07	0.034*
Social Aspects of Dentofacial Deformity (T2)	4 ± 4.25	3.34 ± 5.16	0.109**	4.53 ± 6.31	3.36 ± 4.59	0.424**

*P* < 0.05 is significant p1, Differences between the groups according to the type of surgery

p2, Differences between the groups with skeletal anomalies

Variables are presented as the mean ± standard deviation

*Mann Whitney U test; **Wilcoxon test

## Discussion

This study explored how orthognathic surgery affects the QoL of individuals with dentofacial deformity compared to individuals without dentofacial deformity using the OHIP-14 and OQLQ. All OHIP-14 and OQLQ scores were greater at T1 in all domains in the surgery group than in the control group. After surgery, all the scores decreased significantly in the patient group. Previous studies have reported similar results [26–29]. In systematic reviews and meta-analyses, the authors evaluated the effect of combined orthodontic-surgical treatment on oral health-related quality of life (OHRQoL) and after 6 months, found that condition-specific OHRQoL improved significantly six months after surgery, particularly in terms of perceptions of social aspects and facial appearance [30, 31]. This study showed that orthognathic surgery improved the quality of life of patients with dentofacial deformities.

The scores for functional limitations, physical pain, physical disability, and oral function were significantly greater in the surgery group at T2 than in the control group. In contrast, Su et al. reported that most of the postoperative OHIP-14 scores of patients group were lower than those of the controls. Only the social disability and handicap scores were significantly lower in the patient group. Oral function and awareness of dentofacial aesthetics scores on the OQLQ were greater in the patient group than in the control group [32]. We observed that the surgery group had improved QoL but had lower QoL than did the control group. This can be attributed to orthodontic treatment after surgery. Interestingly, in our study, most scores in the control group increased at T2. This difference can be attributed to differences in awareness, psychological status, and dental problems between T1 and T2. The questions in the survey forms are associated with general problems that everyone can experience. Dentistry students may have developed more awareness about their facial and dental appearance with time. In the literature, such as our study, the control groups mostly consisted of students, but the surveys were applied only once [6, 14, 16, 33, 34]. Our interpretation would have been more appropriate if there had been studies with a second re-evaluation of control group answers.

However, we could not find any results comparing the results at T1 and T2 of the control group in previous studies [10, 32, 35].

Facial aesthetics have an important effect on social life [36] and young people are very active in social life. The mean age of the patients in our study was 24.15 years. Other studies presented similar results related to age [21–23]. Vongkamolchoon et al. [37] reported that the 23 to 30-year-old group exhibited significantly greater scores than in our study. Young patients underwent orthognathic surgery, as shown in our results. Patients with severe dentofacial deformities avoid social interaction and are emotionally unstable [38]; therefore, it is essential to address this problem in young patients.

There were more females than males in our study. Previous studies have reported that females undergo more orthognathic surgeries than males do [23, 33, 39, 40]. In addition, females had better results than males [15, 41]. In contrast, no significant differences were found between males and females [11, 21, 28, 42]. In our study, the scores of the dentofacial aesthetics awareness and social disability domains were significantly greater in females at T1 than in males in the surgery group. This finding indicates that females were more concerned about appearance [43]. Additionally, females had significantly greater scores in the physical pain and physical disability domains at T2 than did males in the surgery group. The findings of Failla et al. [44] can explain this finding in our study. They examined the pain thresholds according to sex and found that females had lower just noticeable pain thresholds.

Class III patients undergo orthognathic surgery more often than Class II patients do [10, 45]. In our study, class III patients constituted the majority of patients. This can be attributed to the fact that Class III patients can have negative opinions about their profile, which is more unattractive than Class II patients can mask their deformity by protruding the mandible [46]. According to the OHIP-14 and OQLQ, Class II patients showed positive results [41]. These results revealed that the dentoskeletal class did not affect QoL [15, 42]. In our study, Class III patients had significantly greater scores at T1 for the OHIP-14 physical pain (*p* = 0.035), social disability (*p* = 0.044), oral function (*p* = 0.026), and social aspects of dentofacial deformity (*p* = 0.034) domains of the OQLQ. At T2, there was no difference in the scores, but QoL improved in both the Class II and Class III groups. Tachiki et al. [8] conducted a study on Class III patients and showed that OQLQ scores for the social aspects of dentofacial deformity, oral function, and facial aesthetics decreased significantly after surgery, but the score for awareness of the dentofacial aesthetics domain did not significantly differ after surgery. According to our results, the patients showed improvements in all domains of the OQLQ, similar to a study in Class III patients [18] Bergamaschi et al. [27] reported that OHIP-14 scores involving social and psychological factors improved; however, the functional limitation domain worsened during the postoperative period, and the physical disability domain did not improve in skeletal Class II patients. This study revealed no significant differences between OHIP-14 scores in skeletal class II patients at T1 and T2, but all scores decreased after surgery in all domains.

Single- or double-jaw surgeries are performed for the treatment of dentofacial deformities. The surgery type did not affect the results of our study, as reported previously [18, 28, 47]. We believe that patients focus on the surgical results of surgeries, not on the operating time or difficulty of the surgery. Patients’ sense of coherence (SOC) may influence their acceptance of surgical results. SOC was explained by sociologist Antonowsky and is a theory of psychosocial factors explaining how well a person manages stress and stays healthy [48]. Kämäräinen et al. [49] evaluated the results of the SOC survey and facial pain, chewing ability, facial appearance, and TMD symptoms in patients who underwent orthognathic surgery after 10–15 years. They found that patients with greater facial appearance improvements were more satisfied. Treatment outcomes have remained constant over time [49].

The strength of this study was that the sample size was large and was defined before the study. The study included a large nonoperated control group with an age range similar to of the patient group. A limitation of our study is the short follow-up duration. The validity of the OQLQ has not been tested in Turkish. Consecutive case selection could have led to selection bias, as the case-control study was conducted without randomization.

## Conclusion

In conclusion, orthognathic surgery improves patients’ quality of life with dentofacial deformities Future studies can incorporate additional surveys to evaluate the aesthetics and satisfaction of each face part.

Long-term longitudinal and multicenter studies are needed for the Turkish population because, as we know, there is no statistical evaluation of orthognathic surgeries throughout the country. We can psychologically evaluate patients before and after the operation, or include a psychologist on the treatment team, as these surveys may not accurately represent the patient as a whole.

## Data Availability

The datasets used and/or analyzed during the current study are available from the corresponding author upon reasonable request.

## Abbreviations

WHO: WHO World Health Organization
QoL: QoL Quality of Life
OHIP-14: OHIP-14 Oral Health Impact Profile-14
OQLQ: OQLQ Orthognathic Quality of Life Survey
SOC: SOC Sense of Coherence
ASA: ASA American Society of Anesthesiologists’
TMJ: TMJ Temporomandibular Joint
